# Improving Cancer Immunotherapy by Targeting the Hypoxic Tumor Microenvironment: New Opportunities and Challenges

**DOI:** 10.3390/cells8091083

**Published:** 2019-09-14

**Authors:** Muhammad Zaeem Noman, Meriem Hasmim, Audrey Lequeux, Malina Xiao, Caroline Duhem, Salem Chouaib, Guy Berchem, Bassam Janji

**Affiliations:** 1 Tumor Immunotherapy and Microenvironment (TIME) Group, Department of Oncology, Luxembourg Institute of Health, Luxembourg, L-1526 Luxembourg City, Luxembourg; 2 Centre Hospitalier du Luxembourg, Department of Hemato-Oncology, L-1526 Luxembourg City, Luxembourg; 3 INSERM U1186, Gustave Roussy Cancer Center, 94805 Villejuif, France; 4 Thumbay Research Institute for Precision Medicine - Gulf Medical University, 4184 Ajman, UAE

**Keywords:** hypoxia, HIF, tumor microenvironment, immune checkpoints, immunotherapy, autophagy

## Abstract

Initially believed to be a disease of deregulated cellular and genetic expression, cancer is now also considered a disease of the tumor microenvironment. Over the past two decades, significant and rapid progress has been made to understand the complexity of the tumor microenvironment and its contribution to shaping the response to various anti-cancer therapies, including immunotherapy. Nevertheless, it has become clear that the tumor microenvironment is one of the main hallmarks of cancer. Therefore, a major challenge is to identify key druggable factors and pathways in the tumor microenvironment that can be manipulated to improve the efficacy of current cancer therapies. Among the different tumor microenvironmental factors, this review will focus on hypoxia as a key process that evolved in the tumor microenvironment. We will briefly describe our current understanding of the molecular mechanisms by which hypoxia negatively affects tumor immunity and shapes the anti-tumor immune response. We believe that such understanding will provide insight into the therapeutic value of targeting hypoxia and assist in the design of innovative combination approaches to improve the efficacy of current cancer therapies, including immunotherapy.

## 1. Introduction 

During the last two decades, the majority of cancer immunotherapies designed by immunologists have mainly focused on potentiating T lymphocyte-mediated anti-tumor adaptive immunity. Strategies based on systemic treatments using interleukin 2 (IL-2) [1] or infusion of autologous T lymphocytes [2] were tested with minor or no therapeutic success, combined with high toxicities. In the last 5 years, immunotherapy approaches using single immune checkpoint blockade (ICB) agents have shown striking clinical efficacy in diverse cancers [3]. ICB-based cancer immunotherapies, notably the T cell immune checkpoint inhibitors anti-cytotoxic T lymphocyte associated protein 4 (CTLA-4) and the anti-programmed cell death protein 1 (PD-1), have provided durable clinical benefit in diverse cancer patients [4]. ICBs hold much promise for the treatment of multiple cancers, including breast, lung, kidney, bladder, prostate cancers, lymphoma, and melanoma [5]. In 2011, the U.S. Food and Drug Administration (FDA) approved ipilimumab (anti-CTLA-4) for the treatment of advanced melanoma, followed by pembrolizumab and nivolumab (anti-PD-1) in 2014. The durable clinical responses observed in patients [4] were largely attributed to the fact that ICB removes inhibitory signals or potentiate stimulatory signals on cytotoxic T lymphocytes rather than targeting tumor cells [4].

However, despite the exciting and promising clinical responses in diverse malignancies, the early promising results obtained using ICB-based monotherapies (anti-CTLA-4, anti-PD-1, or anti-PD-L1) have been seriously compromised by clinical data showing that the majority of ICB-treated patients have incomplete responses and that they fail to achieve higher objective responses. Indeed, the majority of patients treated with ICBs only reaps a short-term benefit or no benefit at all [6]. Briefly, ICB-based therapy is still in its early stages for breast cancer. Both anti-PD-1 and -PD-L1 induce response rates of 19% in patients with heavily pre-treated, chemotherapy-resistant, PD-L1-positive, triple-negative breast cancers (TNBCs) [7]. This clearly shows that the majority of patients (81%) with PD-L1-positive TNBC are completely refractory to ICB, most likely due to tumor adaptation to innate and adaptive immunity. In clear cell renal cell carcinomas (ccRCC), which represent almost 70% of all kidney cancers, it has been reported that the anti-PD-1 nivolumab improved the overall survival and overall objective response rate of patients who had prior antiangiogenic therapy [8]. However, no improvement of progression-free survival was observed with anti-PD-1 therapy [9]. In melanoma, it has also been reported that single treatment with anti-CTLA4 or anti-PD-1 antibodies yielded modest clinical benefit. However, combined therapy with both anti-PD-1 and anti-CTL-A4 significantly improves patient survival [10,11]. 

There is circumstantial evidence indicating that combination approaches improve the efficacy of ICB monotherapies [6]. The efficacy of combination approaches relies on the ability to: (i) increase the tumor infiltration of major cytotoxic immune cells into the tumor bed; (ii) limit the trafficking and function of immune suppressive cells (regulatory T cells, pro-tumoral macrophages and/or myeloid-derived suppressor cells (MDSC)); and (iii) promote the release and presentation of tumor-associated antigens to further potentiate T cell activation. In the design of novel synergistic combinations, we believe that the following questions need to be addressed: (1) What to combine? There is circumstantial evidence suggesting that innovative combination approaches will not be restricted to the use of ICBs, but will include agents that directly target signaling pathways in cancer cells themselves to improve the anti-tumor immune response [12]; (2) How to combine? It has been reported that an increased efficacy was observed when concomitantly inhibiting CTLA-4 and PD-1 pathways relative to inhibition of CTLA-4 or PD-1 alone or sequentially [13]. Therefore, the schedule of administering ICB therapies needs to be carefully considered; and (3) When to combine? It is now well supported that driving a sustained anti-tumor immune response relies on the successful infiltration of immune cells into the tumor bed and the expression of different immune checkpoints and their ligands [14]. 

It should be noted that although combinatorial approaches held considerable promise [4], the potential added risk of toxicity needs to be considered and carefully evaluated in the clinic. Therefore, a safe, robust, and smart combination should be designed in order to extend the clinical benefit of cancer immunotherapy to a larger proportion of cancer patients and tumor types. 

It is also important to keep in mind that an optimal combination strategy should consider the fact that different cells in the tumor microenvironment (tumor cells, stromal cells, and different immune cells) can simultaneously express different levels of diverse immune checkpoints [4,5]. A better understanding of the interactions between the tumor microenvironment and the immune system is crucial for developing new combination strategies. In this review, we will briefly describe the impact of hypoxia as a major tumor microenvironmental factor in regulating the anti-tumor immune response and provide some clues on how targeting hypoxia could improve the therapeutic benefit of cancer immunotherapy.

## 2. The Mechanisms Underlying the Establishment of Hypoxic Tumor Microenvironment and the Impact of Hypoxia on Damping the Anti-Tumor Immune Response 

**Figure 1:**
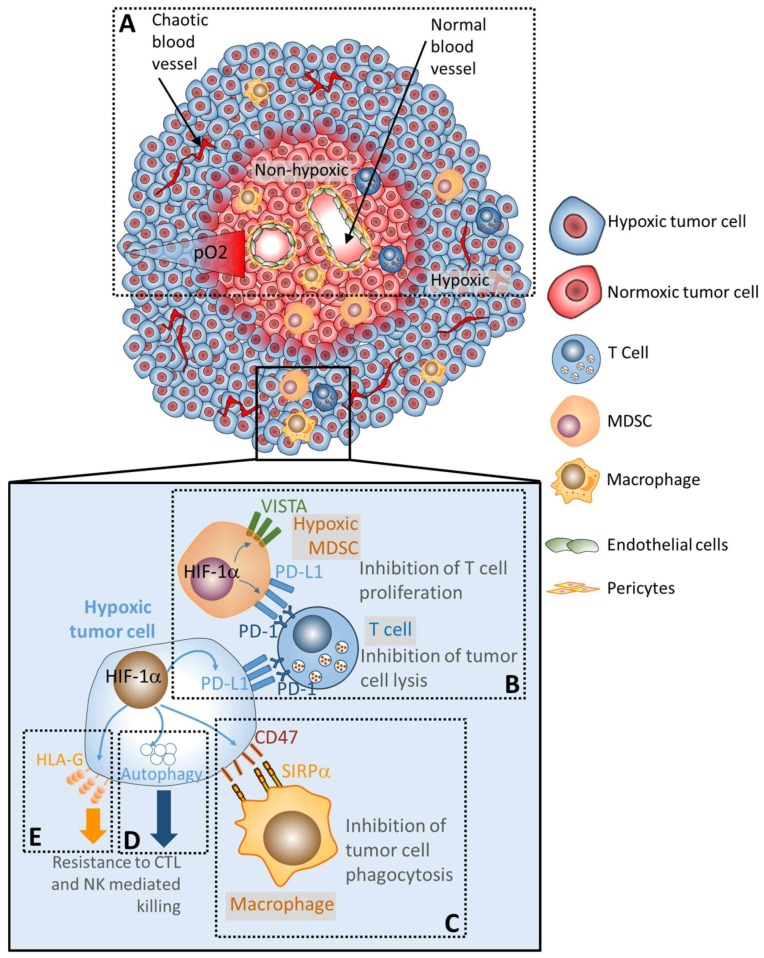
The hypoxic tumor microenvironment and its impact on anti-tumor immunity. (**A**) Hypoxia is established in the tumor microenvironment due to an increase in tumor cell proliferation, and a decrease in oxygen supply. Non-hypoxic tumor regions displayed normal blood vessels covered by well-organized endothelial cells and pericytes. In hypoxic tumor regions, the pressure of oxygen is low which arises from oxygen diffusion limitations due to disorganized, chaotic tumor microvasculature network with leaky vessels. (**B**) Under hypoxia, the stabilization of hypoxia-inducible factor (HIF)-1α in cells upregulates the expression of PD-L1 in hypoxic tumor cells and PD-L1 and VISTA in hypoxic MDSCs. The increased expression of PD-L1 and VISTA results in an inhibition of T cell proliferation and T cell mediated lysis. (**C**) HIF-1α is also involved in the upregulation of cluster of differentiation 47 (CD47) on the surface of tumor cells. Following the binding of CD47 to signal regulatory protein α (SIRPα), expressed on the surface of macrophages, tumor cells provide a strong “don’t eat me signal” to block phagocytosis property of macrophages. (**D**) The activation of autophagy in hypoxic tumor cells impairs tumor cell susceptibility to CTL and NK-mediated lysis by at least two distinct mechanisms involving the degradation of NK-derived Granzyme B and the stabilization of pSTAT3. Other hypoxia-dependent, but autophagy-independent, mechanisms are described in this review including the overexpression of NANOG and miR-210 targeting PTPN1, HOXA1, and TP53I11. (**E**) Hypoxia upregulates the expression of HLA-G on the surface of tumor cells. The upregulated HLA-G binds to ILT2, ILT4 and KIR2DL4 expressed by several immune cells (B and T cells, NK cells, myelomonocytic cells, dendritic cells, monocytes and macrophages) leading to tumor escape from immune surveillance.

Hypoxia in the tumor microenvironment refers to a condition where the pressure of oxygen is lower than 5–10 mm Hg. Therefore, hypoxic regions in the tumors arise from (i) the increase in oxygen consumption due to a marked augmentation in tumor cell proliferation [15], and (ii) the inadequate oxygen supply to the cells and tissues due to the establishment of chaotic tumor microvasculature network with leaky vessels that often fail to rectify the oxygen deficit [16] (Figure 1A). 

The hypoxia-inducible factor (HIF) family of transcription factors are well-defined factors allowing tumor cell adaptation to the hypoxic microenvironment. Three members of the HIF family have been identified: HIF-1α, HIF-2α, and HIF-3α [17]. HIF-1α is a well-described factor involved in the adaptive responses to tissue oxygen level changes [18]. While the expression of HIF-1α in cells occurred in an O_2_-independent manner, its degradation predominantly occurred in an O_2_-dependent mechanism. Under normoxic conditions, HIF-1α is constantly expressed but rapidly degraded by the ubiquitin-proteasome system (in less than 5 min) [19]. However, under hypoxia, the degradation of HIF-1α is blocked, which results in HIF-1α accumulation. The cytoplasmic accumulation of HIF-1α leads to its translocation to the nucleus and the formation of a heterodimer with HIF-1β. Finally, the heterodimer HIF-1α/HIF-1β binds to the hypoxia-responsive element (HRE) in target genes and activates the transcription of several genes involved in various cellular pathways, including autophagy [20].

Hypoxic tumor microenvironment is therefore considered a major mechanism responsible for tumor resistance to several therapies, including chemotherapy and radiotherapy [21,22]. While the major role of hypoxia on chemotherapy and radiotherapy resistance is now well known and extensively reported [23], emerging new data points to hypoxia as a major factor contributing to tumor resistance to immunotherapy [24,25]. This is supported by preclinical and clinical data indicating that the majority of mechanisms overwhelming the antitumor immunity were directly evolved from the hypoxic tumor microenvironment [26]. We and others have reported that hypoxia dramatically impaired the anti-tumor immune response [27,28,29]. Indeed, the hypoxic area of the solid tumor is poorly infiltrated by anti-tumor immune cells. Even if anti-tumor immune cells reach the hypoxic tumor microenvironment, they may not be able to exert their tumor-killing function. Moreover, it has been reported that factors derived from malignant cells participate in the anergic phenotype properties of immune cells in the tumor stroma [30]. Furthermore, anti-tumor immune cells in the tumor microenvironment not only fail to achieve their killing functions but are also co-opted to promote tumor growth [31]. 

## 3. Hypoxia Upregulates the Expression of PD-L1 and Promotes the Establishment of the Immunosuppressive Tumor Microenvironment

PD-1 is an inhibitory receptor expressed on activated T lymphocytes and other immune cells [32]. The expression of PD-1 leads to T cell exhaustion following its binding to two ligands, programmed death ligand 1 and 2 (PD-L1 and PD-L2). Thus, the interaction between PD-1 and its ligands provides a negative signal to T cells, which ultimately blocks their cytotoxic functions [4]. The molecular mechanism(s) underlying the expression of PD-L1 in different tumor types have been extensively investigated. Hypoxia, via HIF-1α, directly up-regulates the expression of PD-L1 in various tumor cells (melanoma, lung, breast and prostate cancer) by directly binding the HRE in the promoter of PD-L1 gene [33,34]. HIF-2α is also involved in PD-L1 induction in ccRCC. The mutation of the *VHL* gene induced HIF-2α stabilization in ccRCC cells. Stabilized HIF-2α led to the upregulation of PD-L1 in vitro. Furthermore, in ccRCC patients, the mutation status of VHL was associated with HIF-2α stabilization. Such stabilization was strikingly correlated with an increased expression of PD-L1 [35]. In immune cells, such as MDSCs and macrophages, HIF-1α selectively upregulates the expression of PD-L1. MDSCs displaying high expression levels of PD-L1 negatively impact the functions of cytotoxic T lymphocytes (CTL). Blocking PD-L1 abrogated MDSC-mediated T cell suppression [33,36] (Figure 1B). 

## 4. Hypoxia Induces the Expression of the Immune Checkpoint V-Domain Ig Suppressor of T Cell Activation (VISTA) and Promotes the Immunosuppressive Function of Tumoral MDSC

In addition to PD-L1, it has been recently shown that VISTA is overexpressed in the hypoxic areas of colon cancer patients and CT-26 colon mouse model [37]. Indeed, VISTA was preferentially expressed on myeloid cells, namely CD11b^high^ CD11c^+^ dendritic cells, CD11b^high^ F4/80^+^ macrophages, with the highest expression on CD11b^high^Gr1^+^ MDSCs infiltrating the hypoxic areas of the tumor (Figure 1B). The infiltration of MDSCs from the periphery to the hypoxic area of the tumor is associated with the hypoxia-dependent increase in the expression of stromal-derived factor 1 (SDF1, CXCL12) [38]. Furthermore, the upregulated expression of VISTA under hypoxia was attributed to the ability of HIF-1α, but not HIF-2a, to bind to the VISTA promoter. The functional consequence of hypoxia-dependent induction of VISTA is the suppression of T cell proliferation and activity [39]. 

## 5. Hypoxia Upregulates the Macrophage Immune Checkpoint CD47 “Don’t Eat Me Signal” and Induces Tumor Cell Escape from Phagocytosis 

Cluster of differentiation 47 (CD47), also known as integrin-associated protein, is a transmembrane immune checkpoint protein expressed on the cell surface of tumor cells and hematopoietic cells [40]. Following the binding of CD47 to its ligands—signal regulatory protein α (SIRPα) and thrombospondin-1 (TSP-1)—on the surface of macrophages and dendritic cells, CD47 provides a robust “don’t eat me signal” to block phagocytosis [41] (Figure 1C). The elevated expression level of CD47 is an adverse prognostic factor in acute myeloid leukemia [42]. Targeting CD47 for cancer therapy has sparked great interest. Clinically, the use of anti-CD47 5F9 appears to be safe and well tolerated in most patients. However, it should be highlighted that the most significant side effects of 5F9 are transient anemia, fatigue and headache. Mechanistically, very little is known about the molecular mechanisms underlying the transcriptional regulation of the CD47 gene. Nevertheless, several signaling pathways, transcription factors [43,44], and miRNA [45] have been reported to regulate the expression of CD47. 

Several ICBs are currently being developed to specifically target and activate different innate immune cells, including macrophages and dendritic cells (DCs) [42,46]. Blockade of the CD47 “don’t eat me signal” using monoclonal antibodies against CD47 increases macrophage-mediated phagocytosis and elimination of various solid tumors [41]. When using several tumor models syngenically transplanted into immune-competent mice, blocking CD47 promotes massive destruction of tumor cells by a mechanism mainly depending on T lymphocytes activation [47]. Human CD47-blocking monoclonal antibodies have incredible efficacy in numerous patient-derived xenograft (PDX) preclinical models of breast, lymphoma, bladder, colon, glioblastoma, lung, acute lymphocytic leukemia, and acute myeloid leukemia [41,48,49]. CD47 blockade is, therefore, a novel validated target for macrophage-mediated ICB-based cancer immunotherapy. 

Induction of phagocytosis by anti-CD47 blockade results in increased antigen uptake and presentation, thereby simultaneously enhancing innate and adaptive immune systems [50]. CD47 blocking therapy will, therefore, synergize with immune checkpoint inhibitors that target the adaptive immune system. Previous studies have established that both innate and adaptive immune systems are required for the complete therapeutic response of ICBs [51,52,53]. 

In breast cancer, evidence has been reported that hypoxia positively regulates the expression of CD47 by showing that the expression of CD47 is positively correlated with the expression of HIF-1α downstream target genes [44]. In triple-negative breast cancer cells, HIF-1α induced the expression of CD47, leading to cancer stem cell phenotype switch and cancer cell escape from phagocytosis, which was mediated by bone marrow-derived macrophages [44]. In pancreatic adenocarcinoma, hypoxia also upregulated the expression of CD47, thus blocking the pro-phagocytic signals in both MDSC and macrophages [54,55]. 

## 6. Hypoxia-Induced Autophagy Impairs Tumor Cell Susceptibility to Immune Cell Attack

Autophagy is a cellular pathway involved in the degradation of cellular components, including damaged organelles and misfolded proteins in the lysosomal compartment. Such degradation provides nutrients to maintain cellular functions under stress conditions, such as hypoxia [56]. Although the activation of autophagy by hypoxia in tumor cells can occur either in a HIF-1- dependent or HIF-1-independent manner, the major negative impact of hypoxia-induced autophagy on the anti-tumor immunity involves HIF-1α. Briefly, HIF-1α induces the expression of the Bcl-2 homology (BH) 3-only protein Bcl-2/adenovirus E1B 19 kDa-interacting protein 3 (BNIP3) and the related protein, BNIP3L [57]. Under hypoxia, the BNIP3/BNIP3L complex activates autophagy by preventing the association between Beclin1 (BECN1) and B-cell lymphoma 2 (Bcl-2) [58]. We have previously reported that the susceptibility of lung cancer cells to CTL-mediated killing was dramatically impaired under hypoxia through the activation of autophagy. We show that inhibiting autophagy genes Beclin1 or ATG5 restored lung cancer cell susceptibility to CTL mediated lysis under hypoxic stress. The molecular mechanism underlying the restoration of CTL-mediated killing of lung cancer cells following autophagy blockade is related to the ability of tumor cells to induce ubiquitin–proteasome system (UPS)-dependent degradation of phospho-signal transducer and activator of transcription 3 (pSTAT3) [59,60]. In addition to the mechanism described above, hypoxia, via HIF-1, upregulates the expression of the stem cell self-renewal transcription factor Nanog Homeobox (NANOG) at both transcriptional and translational levels. HIF-1α silencing is sufficient to downregulate hypoxia-dependent induction of NANOG. Genetic silencing of hypoxia-induced NANOG in tumor cells restored CTL-mediated tumor cell killing. The molecular mechanisms underlying NANOG-dependent inhibition of CTL-mediated killing involve STAT3 phosphorylation and its nuclear translocation as well as autophagy activation [61,62,63]. Furthermore, HIF-1α also impairs the tumor cell susceptibility to CTL-mediated killing by inducing the expression of microRNA (miR)-210, which targets the non-receptor protein tyrosine phosphatase type 1 (PTPN1), homeobox A1 (HOXA1), and tumor protein p53-inducible protein 11 (TP53I11) [64]. 

The negative impact of hypoxia is not only restricted to the impairment of cancer cell susceptibility to CTL-mediated killing but also applies to Natural Killer cell (NK)-mediated killing. Evidence has been reported that hypoxia is involved in the shedding and in the downregulation of major histocompatibility complex (MHC) class I polypeptide-related sequence A (MICA), a ligand for the activating NKG2D receptor expressed on the surface of cancer cells. The shedding of MICA led to tumor cell escape from NK- and CTL-mediated killing [65,66,67]. 

Moreover, hypoxic tumor cells take advantage of the induction of autophagy to selectively degrade the serine protease granzyme B (GZMB) released by NK cells. The release of cytotoxic granules containing perforin (PRF1) and GZMB by NK cells is one of the major mechanisms responsible for tumor cell killing by NK cells. These cytotoxic granules enter target tumor cells by endocytosis and traffic to large specific endosomes named “gigantosomes”. Evidence has been reported that the proapoptotic protein GZMB is selectively degraded by the activation of autophagy in hypoxic cells, thus inhibiting NK-mediated killing of cancer cells (Figure 1D). The mechanism by which autophagy selectively degrades granzyme B is not yet fully understood [68,69].

## 7. Hypoxia Upregulates the Expression of the Non-Classical and Immunosuppressive MHC Class I (HLA-G) 

HLA-G is a non-classical MHC-I molecule expressed in several tumor types including melanoma, glioblastoma, colorectal, ovarian and cervical tumors. HLA-G expression in tumors was related to advanced tumor stages, poor prognosis [70,71] and immune suppression [72,73]. The immunosuppressive functions of HLA-G relies on its ability to bind to ILT2, ILT4, and KIR2DL4 expressed by several immune cells, including B cells, T cells, NK, myelomonocytic cells, dendritic cells, monocytes, and macrophages [74,75] (Figure 1E). HLA-G is therefore proposed as an immune checkpoint [76] and an attractive therapeutic target [77]. Several HREs have been identified in the promoter of HLA-G [78,79], indicating that the hypoxia-dependent mechanism underlying the expression of HLA-G most likely relies on the ability of HIF-1 to bind to HRE motifs and induce *HLA-G* transcripts. 

## 8. The Challenges and Opportunities of Targeting Hypoxia 

Our understanding of the molecular mechanisms underlying intratumoral hypoxia has largely fueled interest in the development of strategies to inhibit hypoxia in cancer therapy. However, the excitement for developing such strategies has been tempered by the lack of selectivity of HIF-1 inhibitors that might be thought of as a gold standard. Obviously, the unique opportunity for exploiting hypoxia inhibitors in the clinic is their validation in appropriate preclinical models and more importantly, in early clinical trials. Targeting hypoxia could be achieved using hypoxia-activated prodrug or drugs that directly or indirectly modulate HIFs. A hypoxia-activated prodrug is an inactive compound that can be converted to a pharmacologically active drug in hypoxic cells or tissues. The conversion is achieved by one electron cellular reductases to generate prodrug radical that can be further reoxidized to the initial prodrug in a non-hypoxic cell. The prodrug can also be converted (in a single step) into a cytotoxic drug via the two-electron reduction pathway [80]. The hypoxia prodrug tirapazamine and its analog SN30000, as well as TH-302 and apaziquone EO9, have been evaluated in clinical trials with some disappointments. However, the published results of the combination of TH-302 with gemcitabine in pancreatic cancer [81] or with doxorubicin in soft tissue sarcoma [82] in phase II clinical trials are encouraging. Recently, it has been reported that TH-302 significantly reduced hypoxia in a preclinical mouse prostate model. In addition, combining TH-302 therapy with T cell immune checkpoint blockades CTLA-4 and PD-1 cured more than 80% of tumors in a mouse prostate-derived model, most likely by driving an influx of T cells into hypoxic zones and by reducing both MDSC and granulocytic subsets in the microenvironment [83]. Although the clinical trials using EO9 were negative, the loco-regional administration of this drug in patients having superficial bladder cancer has shown efficacy. Based on these data, two phase III clinical trials (NCT00598806 and NCT00461591) have been performed using EO9 as adjuvant therapy in bladder cancer patients treated with surgery. 

According to their mode of action, an increasing number of HIF modulating drugs are being reported. These drugs can be classified as modulators of the expression, translation, degradation, DNA binding, and transcriptional activity of HIF-proteins. Although several excellent reviews have provided a comprehensive overview of all drugs affecting hypoxia and detailed their mode of action [23,84,85,86], here we will summarize the major drugs modulating the expression of HIFs according to the three mechanisms of action described above. 

The drugs directly modulating HIF-mRNA include HIF-1α antisense oligonucleotides EZN-2698 [87], aminoflavone [88], and thioredoxin inhibitors (AJM290 and AW464) [89], although these particular drugs are also reported to stabilize HIF-1α and HIF-2α. Several drugs targeting signaling pathways involved in the control of HIF-1α mRNA translation have been identified, such as inhibitors of the PI3K/AKT/mTOR pathway [90,91,92]; topoisomerase 1 inhibitors (Irinotecan and Topotecan) [93,94]; and methoxyestradiol (2ME2) [95]. Drugs that induced HIF-1α degradation include Geldanamycin analog 17-AAG (tanespimycin) and 17-DMAG (alvespimycin) [96]; the next-generation EC154 molecule [96]; HDAC inhibitors (vorinostat, romidepsin, panobinostat, and belinostat, which might also act as inhibitors of HIF-1α translation) [97]; and the melphalan-derived alkylating agent PX-478 [98]. Drugs inhibiting the transcriptional activity of HIFs were also reported, such as FM19G11 [99], acriflavine [100], and PT2385, which inhibits the transcriptional activity of HIF-2α. Finally, chetomin has been reported to disrupt HIF-p300 interaction, thereby inhibiting HIF–DNA binding activity [101]. 

## 9. Concluding Remarks

While the hypoxic tumor microenvironment has long been considered as one of the most attractive targets in cancer drug development, numerous strategies and drugs described in this review have been suggested to target hypoxic tumor cells, including hypoxia-activated prodrugs, small HIFs inhibitors, or drugs targeting HIF-downstream signaling pathways [85,102,103]. Despite considerable efforts intended to bring hypoxia inhibitors to the clinic, there are so far no approved drugs that directly target hypoxia or HIF-dependent pathways, and the results from the various clinical trials have been mostly disappointing [84,104]. This could be, at least in part, attributed to the ability of HIF to control a highly complex network connecting several signaling pathways and various overlapping mechanisms in tumor cells and other cells in the tumor microenvironment. In addition, the lack of specificity of the majority of HIF inhibitors impedes the efficacy of these drugs and participates in the failure of clinical trials designed to include HIF-1 inhibitors. Nevertheless, recent discoveries showing that hypoxia negatively impacts the tumor immune response by modifying the expression of main immune checkpoints (e.g., PD-L1, CD47, PD-1, HLA-G, …) provide a major opportunity for innovative combination approaches. Such combinations might pave the way for setting up new strategies for therapeutic intervention to enhance the clinical benefit of immune checkpoint blockades in solid tumors. Future efforts should be focused on the development of potent and selective inhibitors of hypoxia, which is considered among the greatest challenges in cancer drug development.
